# Microstructure–Property Relationship of Polyurethane Foams Modified with Baltic Sea Biomass: Microcomputed Tomography vs. Scanning Electron Microscopy

**DOI:** 10.3390/ma13245734

**Published:** 2020-12-16

**Authors:** Paulina Kosmela, Jan Suchorzewski, Krzysztof Formela, Paweł Kazimierski, Józef Tadeusz Haponiuk, Łukasz Piszczyk

**Affiliations:** 1 Department of Polymer Technology, Faculty of Chemistry, Gdansk University of Technology, G. Narutowicza 11/12, 80-233 Gdansk, Poland; jozhapon@pg.edu.pl (J.T.H.); lukpiszc@pg.edu.pl (Ł.P.); 2 Division Built Environment, Department Infrastructure and Concrete Structures, Material Design, RISE Research Institutes of Sweden, Brinellgatan 4, 501-15 Borås, Sweden; jan.suchorzewski@ri.se; 3 Department of Concrete Structures, Faculty of Civil and Environmental Engineering, Gdansk University of Technology, G. Narutowicza 11/12, 80-233 Gdansk, Poland; 4 Institute of Fluid Flow Machinery, Fiszera Str. 14, 80-231 Gdansk, Poland; pkazimierski@imp.gda.pl

**Keywords:** rigid polyurethane foam, biopolyols, microcomputed tomography, scanning electron microscope, microstructure, pores, sustainable materials

## Abstract

In this paper, novel rigid polyurethane foams modified with Baltic Sea biomass were compared with traditional petro-based polyurethane foam as reference sample. A special attention was focused on complex studies of microstructure, which was visualized and measured in 3D with high-resolution microcomputed tomography (microCT) and, as commonly applied for this purpose, scanning electron microscopy (SEM). The impact of pore volume, area, shape and orientation on appearance density and thermal insulation properties of polyurethane foams was determined. The results presented in the paper confirm that microcomputed tomography is a useful tool for relatively quick estimation of polyurethane foams’ microstructure, what is crucial especially in the case of thermal insulation materials.

## 1. Introduction

Polyurethane foam has multiple applications in water sports and other marine industries, carpentry and as sleeping mattress but mostly are used as thermal insulation. It is the world’s sixth most used polymer with an annual production of 18 million tons [1]. Polyurethanes are one of the most widely used thermal insulation materials in buildings due to their very low thermal conductivity and relatively good mechanical performance in comparison with other popular thermal insulators like polystyrene and mineral wool. The standard value of thermal conductivity factor for PU foams is equal to *λ* = 0.020–0.027 W/m^2^K, while the compressive strength σ_c_ is between 100–500 kPa [2,3]. PU foams have disadvantages, like high vapor diffusion resistance factor μ = 50–100 and low fire resistance (up to 120 °C) [2,3]. The insulation materials, like polyurethanes, play an important role in the building industry as they determine long-term energy efficiency and sustainability.

In polyurethane industry, the use of renewable or waste materials, mainly various compounds containing hydroxyl groups like vegetable oils, which are commonly used in the formulation of polyurethane products. Depending on the geographical location, different types are used: rapeseed and sunflower oil in Europe, palm and coconut oil in Asia, and soybean oil in the North America [4,5,6]. Vegetable oils, which are currently one of the raw materials used in the production of polyols, dedicated for the production of polyurethane materials, are mainly basic components of the food industry. The introduction of oils as for the large-scale production of plastics could increase their prices and increase the costs of various types of food products. Therefore, the use of byproducts in the processing of renewable raw materials, such as biomass or waste glycerol, is currently gaining more attention [7,8,9]. During production of one ton of biodiesel, 90–110 kg of waste glycerol is produced with varying chemical composition [10]. Due to a large amount of produced byproduct in the form of glycerol waste, diverse solutions for its sustainable management have been developed [11,12]. Biomass is another example of a raw material used in the plastics industry. The number of reports on the use of various types of the biomass, not only as a source of renewable energy but also as a raw material for receiving, among others, rigid polyurethane foams is constantly increasing [13,14,15].

The most important renewable sources used in the plastics industry include biomass from wood, arable crops, and aquatic plants [16]. Potential biomass that can be used in the plastics industry is biomass from the aquatic environment (flowering plants and algae inhabiting water reservoirs). Biomass of algae contains large amounts of carbohydrates and small amounts of lignin, which makes algae an excellent candidate for the synthesis of bioplastics [17,18]. The use of algae for bioplastics can be carried out directly or indirectly. Direct methods include mixing polymers with algae biomass [19,20,21,22], and indirect methods use algae biomass to obtain a component for the synthesis of bioplastics [23,24,25,26,27,28,29].

The chemical modifications affect the PU foams microstructure and key macroscopic performance like thermal conductivity and mechanical strength. As the pore diameters in PU foams are very fine (d = 0.02–0.06 mm) it is extremely difficult to evaluate precisely their distribution with commonly applied methods like scanning electron microscopy and optical microscopy [30]. Recently, the application of high-resolution X-ray microcomputed tomography (microCT) has increased significantly. MicroCT is a nondestructive 3D imaging technique. The basic physical X-ray principal of computed tomography is the interaction of ionizing radiation with the material, where the so-called photoeffect builds the main interaction mechanism. As an X-ray beam penetrates an object, it is exponentially attenuated according to the material along its path. The energy-dependent material constant appearing in the exponent of this attenuation formula is called the linear attenuation coefficient. It expresses the amount of radiation that is attenuated on an infinitely small distance, in which the final attenuation reflects the sum of all these local linear attenuations along the X-ray beam. Therefore, an X-ray projection (or X-ray image) represents an image of the sum of all local attenuations along the X-ray beam. The 3D images of the interior of an object are obtained by collecting a series of 2D images that are stored while the sample is rotated. With the development of microCT, complementary techniques as well as new image processing algorithms and analysis techniques have evolved. These unique techniques opened the field of microCT to many new applications. Imaging techniques with image analysis offer the possibility to analyze the size and shape of each individual particle present in a sample. In these methods, touching particles are separated, so that each individual particle can be identified, counted and measured. The powerful imaging capabilities of X-ray tomography are now available in a range instruments suitable for microscopic imaging of small objects with a wide range of applications such as 3D biomedical [31,32], food studies [33], soft tissue research [34,35], insects and fishes, microelectronics, geological studies [36,37] including porous rocks for oil recovery purposes, cementitious materials [38,39,40] and wood structure after pyrolysis [41]. X-ray microcomputed tomography has been recently often used as a powerful tool for analysis of polyurethane foams. Patterson et al. [42] were particularly interested in compressive and tensile response of PU foams and differences in molding shapes (block and conical). They investigated deformed specimens, finding shear band zones connected usually with presence of larger pores. PU foams modified with glass microspheres (syntactic foams) structure were investigated by Adrien et al. [43]. They found strong dependency of failure type on matrix type (soft or rigid foam). For PU soft matrix, the hollow microspheres (pore like structures) with lower radius and skin thickness were mainly broken in compression, while for rigid PU matrix their exhibit opposite behavior. McDonald et al. [44] performed one of the first microCT scans of polymeric conventional and auxetic PU foams under load (uniaxial tension) analyzing their internal structure evolution. They observed severe changes in the foam density and internal structure. Similar study was performed for PU foams by Youssef et al. [45], where the internal foams microstructure was exported to FEM 3D model. The clear influence of pore structure on material failure was observed. With use of microCT, it was proven that natural origin additives (oak quercus robur bark) decrease the water absorption, thermal conductivity, brittleness, aging in PU foams with low cost by changing the pore structure [46].

The main objective of this paper is a comprehensive investigation of novel rigid polyurethane foam modified with Baltic Sea biomass (biopolyol from liquefied marine environment biomass: *Enteromorpha* and *Zostera marina*) as a possible sustainable material for building industry. A 3D microstructure analysis with a high-resolution microcomputed tomography system (microCT) of modified foams in comparison with traditional petro-based polyurethane was performed. Moreover, microstructure was also examined using SEM in order to comparison with microCT analysis. The influence of pores volume, area, shape and orientation on apparent density and thermal insulation properties were evaluated.

## 2. Materials and Methods

### 2.1. Materials

Rigid polyurethane (PU) foams were obtained using the biopolyol LB (liquefaction biopoliol) synthesized in our laboratory and Rokopol^®^RF551 petrochemical polyol (a polyoxyalkylene multihydroxyls alcohol) from PCC Group. The selected properties of polyols are shown in Table 1. The biopolyol LB was obtained in the biomass liquefaction process, which included: 10 wt.% macroalgae of *Enteromorpha* and 90 wt.% of seagrass (*Zostera marina*), originating in the Baltic Sea. As a solvent for the liquefaction process, purified waste glycerol containing 99% obtained from the company Bio-Chem Sp. z o.o. (Olszanka, Poland) and poly(ethylene oxide) (PEG400) from Avantor Performance Materials Poland S.A. (Gliwice, Poland) were used. During the liquefaction, sulfuric acid (VI) as a catalyst and sodium hydroxide (both reagents from Avantor Performance Materials Poland S.A. (Gliwice, Poland) were used to neutralize biopolyols. The detailed information about reaction conditions are presented elsewhere [47]. As an isocyanate component, the polymeric 4,4′-methylene diphenyl diisocyanate (pMDI) with a free NCO content of 31.5% from BASF (Ludwigshafen, Germany) was applied. The catalysts used were: 33 wt.% potassium acetate solution in ethylene glycol—PC CAT^®^ TKA30 from Performance Chemicals (AC) (Buchholz in der Nordheide, Germany), 75% wt. potassium octoate solution in diethylene glycol—Dabco K15, 33 wt.% solution of triethylenediamine in dipropylene glycol—Dabco33LV from Air Products (Allentown, PA, USA and dibutyltin dilaurate (DBTDL) from Sigma Aldrich (Saint Louis, MO, USA). Tegostab B 8465 from Evonik Industries AG (Essen, Germany) was used as a stabilizer of the porous structure (SPC), and *n*-pentane from Sigma Aldrich (Saint Louis, MO, USA) was used as a blowing agent. Tris(chloropropyl) phosphate (TCCP) was also added as a flame retardant, which further reduced the viscosity of the polyol mixture from LANXESS Deutschland GmbH (Koln, Germany).

### 2.2. Synthesis of Rigid PU Foams

Rigid PU foams were obtained using the one-step method from a two-component system. The isocyanate index (I_ISO_) has been selected based on literature reports [48,49,50] and it was 200 or 300. Component A was a polyol mixture consisting of Rokopol^®^RF551 polyol and biopolyol LB, catalysts, surfactant, flame retardant and a blowing agent. Component B was an isocyanate. Foam formulations are presented in Table 2. Both components were mixed in a polypropylene cup with a mechanical agitator at a speed of 2000 rpm, poured into an open mold and heated for 24 h at 60 °C.

### 2.3. Methods

#### 2.3.1. Apparent Density

The apparent density was determined in accordance with ISO 845. The mass of the samples was determined using an analytical balance with an accuracy of 0.1 mg, and the volume was determined after dimensioning the rolls using an electronic caliper with an accuracy of 0.1 mm.

#### 2.3.2. Thermal Conductivity

The thermal conductivity of the foams was tested in accordance with ISO 8301 standard using the Holometrix 2300. The average temperature of analysis was 10 °C, temperatures of lower and upper plates 0 and 20 °C, respectively. Three samples with size of 300 × 300 × 50 mm were tested for each composition. Thermal resistance is closely related to the conductivity coefficient (*λ*) and depends on the thickness of the material (*d*), as presented in Equation (1):

(1) $$R=\;\frac{d}{\lambda }\;$$

#### 2.3.3. Scanning Electron Microscopy

The cell morphology of polyurethane samples was investigated with a Quanta FEG 250 environmental scanning electron microscope (SEM) (FEI Company, Hillsboro, OR, USA) using an acceleration of 25 kV. The average value of pore diameter was calculated based on SEM images in ImageJ. The result was obtained from averaging 100 measurements.

#### 2.3.4. Microcomputed Tomography

To study the internal structure of polyurethane foams and its relation with global material properties each specimens with diameter *d* = 15 mm and height *h* = 15–25 mm were scanned in microCT. Specimens were scanned by means of the 3D X-ray microtomograph Skyscan 1173 (Bruker, Kontich, Belgium) [51,52]. X-ray microtomography (called microCT or µCT) is a 3D imaging technique which uses X-rays to create cross-sections of a physical object that is used to recreate a virtual model (3D model) without destroying the original object. The X-ray microtomograph used in this research represents a new generation in high-resolution desktop X-ray microtomography systems [38,51]. The scans were completed to ten times faster with the same resolution and image quality as compared to previous microCT with a fixed source-detector design. The scanner was equipped with the newly developed 130 keV microfocus X-ray source with a very stable focal spot position and flat panel sensor of a large format (5 Mpx) with a special protection by a lead-glass fiber-optic window. As compared to usual X-ray microtomographs, this scanner has two basic advantages: (a) large specimens up to 150 mm in diameter may be scanned and (b) specimens are scanned with a higher precision (2–3 microns).

**Figure 1:**
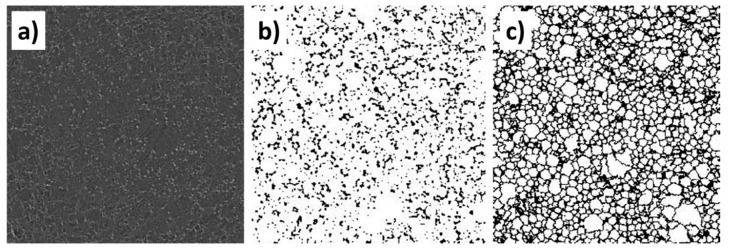
Microtomography (microCT) scan postprocessing: (**a**) reconstructed scan, (**b**) scan after threshold and (**c**) image after anisotropic diffusion.

The specimens were scanned with resolution of 9 μm, by rotation of 180°. An X-ray voltage energy of 35 keV and current of 175 μA and exposure time of 500 ms was found to give sufficient contrast for the weakly absorbing polymeric foam material. The wavelength profile of the beam was shaped using no filter, placed just in front of the exit window of the X-ray source. The specimen was scanned with rotation step of 0.2° for high image precision. Moreover, frame averaging 4 and random movement 10 was used. Then the scans were reconstructed into 3D tomographical volumes using Skyscan Nrecon 1.6.9.8 software (Bruker, Kontich, Belgium), with ring artefact correction at level of 8 and smoothing level 6. Reconstructed images were analyzed in Skyscan CTAn 1.17.7.2 software(Bruker, Kontich, Belgium), and all steps of analysis are summarized in Figure 1. At first step, the images filtering function was used to sharpen the images (Figure 1a). Then, a threshold of 60–255 was applied (Figure 1b). The technical difficulties in microCT scanning of low-density foams, like polyurethane, were reported due to its relatively low attenuation coefficient [52]. These difficulties may be overcome by specimen preparation with contrast agents [52] or with image postprocessing [53]. In this paper the second approach was chosen. The option of watershed operations of anisotropic diffusion was used to reassemble broken objects (pores) (Figure 1c) in the image. At the end black and white pixels smaller than 25 μm was applied. The volume used for analysis was limited to *d* = 10 mm and *h* = 10 mm, due to very high analysis time of micropores. The whole specimens consisted of around 1,500,000 pores.

## 3. Results and Discussion

### 3.1. Microcomputer Tomography

**Figure 2:**
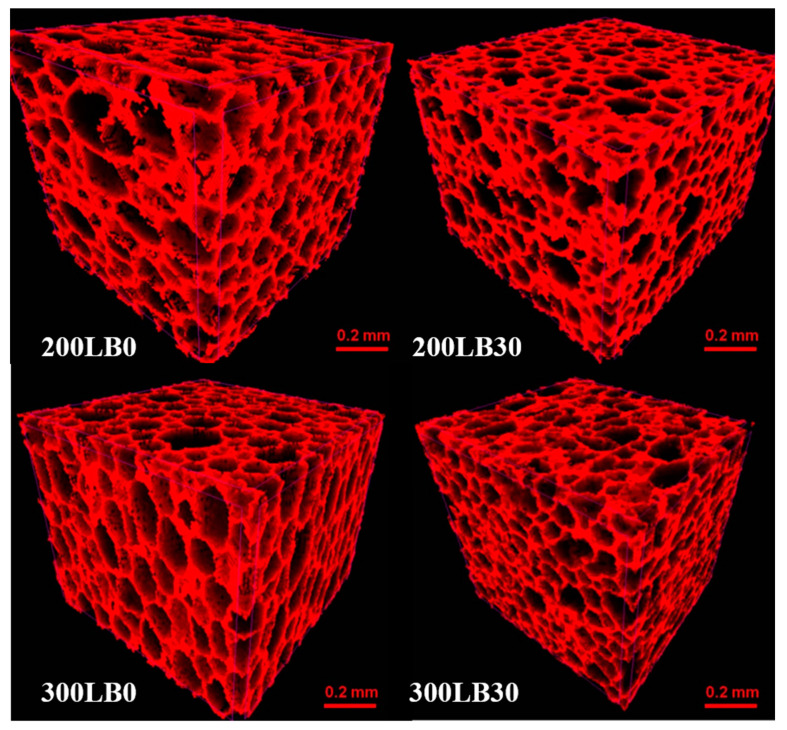
3D images of foams skeletons and pores for PU foams.

The internal structure of specimens was analyzed both qualitatively and quantitatively. Based on the preliminary results, in this work, the most differing specimens coded as 200LB0, 200LB30 and 300LB0, 300LB30 were investigated. The images of 3D internal structure for I_ISO_ = 200 and I_ISO_ = 300 with different amounts of biopolyols are presented at Figure 2. Depending on the type of foam, the increase in the isocyanate content resulted in the formation of pores with a less spherical shape (for foams with 0 wt.% biopolyol) and a more spherical shape (for foams with 30 wt.% biopolyol). The addition of biopolyols from sea biomass caused a decrease of pore size for both isocyanate contents, while the apparent density remained constant and thermal conductivity decreased (see Table 3). A similar microstructural effect of pore diameters decrease was observed when other organic biopolyols (from oak bark) were added to polyurethane foams [46].

**Figure 3:**
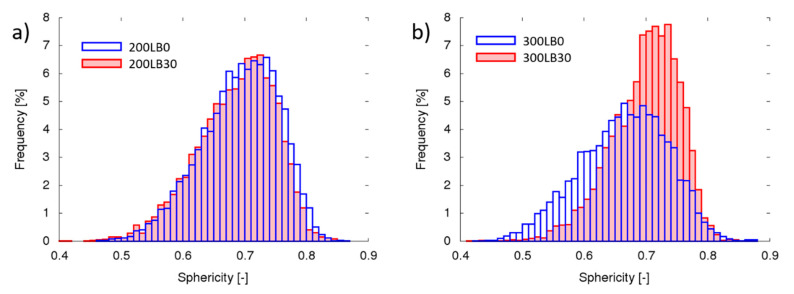
Pore sphericity for samples prepared with: (**a**) I_ISO_ = 200 and (**b**) I_ISO_ = 300.

**Figure 4:**
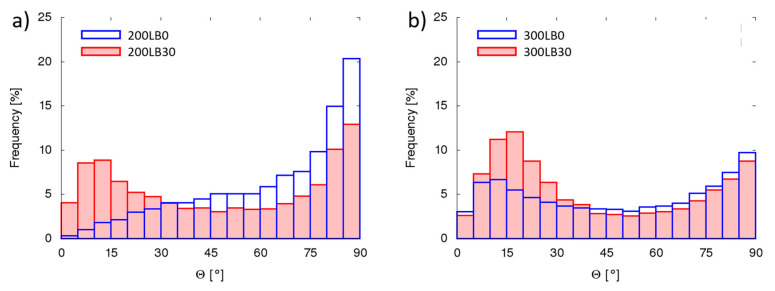
Pores vertical inclination angle *Θ* for (**a**) I_ISO_ = 200 and (**b**) I_ISO_ = 300.

**Figure 5:**
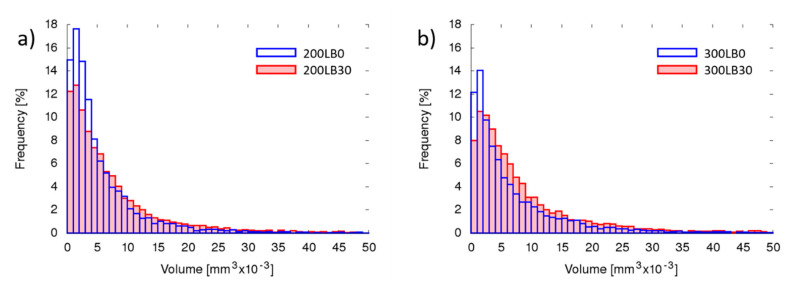
Pores volume for (**a**) I_ISO_ = 200 and (**b**) I_ISO_ = 300.

**Figure 6:**
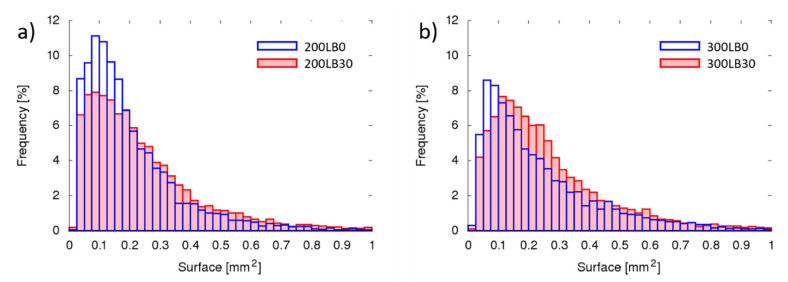
Pores’ external surface area for (**a**) I_ISO_ = 200 and (**b**) I_ISO_ = 300.

The pores’ geometrical properties in each specimen were measured considering parameters like pore sphericity (Figure 3), orientation (inclination angle) (Figure 4), volume (Figure 5), and surface (Figure 6).

The sphericity *ψ* is a dimensionless parameter describing how closely the analyzed object approaches the mathematically ideal sphere and can be calculated with the following equation (Equation (2)):

(2) $$\Psi =\frac{\pi^{\frac{1}{3}}\times {\left(6\times V_{p}\right)}^{\frac{2}{3}}}{A_{p}}\;$$

where *V_p_* represents object volume, *A_p_* is the object surface. Therefore, for perfect sphere sphericity *ψ* = 1, for tetrahedron *ψ* = 0.671 and for infinite straight line *ψ* = 0 [54]. The sphericity measured for foam cells with I_ISO_ = 200 was similar with and without biopolyol and was scattered from *ψ_min_* = 0.40 to *ψ_max_* = 0.86 with average *ψ_avg_* = 0.72, whereas for I_ISO_ = 300 in similar range the distribution with and without biopolyol was different (Figure 3). For foam LB = 30% average sphericity was *ψ_avg_* = 0.74 and without biopolyol admixtures *ψ_avg_* = 0.69. Moreover, the pores in foam with biopolyol had less concentrated distribution of sphericity (over 66% of pores had sphericity in the range 0.69 < *ψ* < 0.77 for LB = 30%, while for LB = 0% 0.58 < *ψ* < 0.75).

Many works report anisotropic behavior of polyurethanes, with usually greater mechanical strength in direction of foam growth [43,44,45]. This phenomenon is connected with internal structure. As the foams grow in the form vertically, they usually have an oval shape (elliptical in section) with the semimajor axis in vertical direction. The following phenomenon may be measured in microCT scans as the orientation of the object’s major axis inclination to vertical axis Θ. Angle 0° means that the major axis (basing on the moment of inertia) is perfectly vertical, while 90° means fully horizontal. For foams with I_ISO_ = 200, the biopolyol addition caused more equal orientation distribution with peaks in vertical (25%) and horizontal (29%) orientation, while without biopolyol 47% of pores where oriented horizontally between 75–90°. This behavior may be the result of horizontal interconnection of single pores. For foams with I_ISO_ = 300 the presence of biopolyol caused again more smooth distribution of orientation, while original polyurethane had pores with vertical orientation (47% in the range 0–30°). The result clearly corresponds with sphericity (Figure 3) and the internal structure images (Figure 2).

The number of fine pores was greater in foams with I_ISO_ = 200, than in I_ISO_ = 300 (Figure 5). The biopolyol addition caused slightly more equal distribution of volumes, with less fine pores (V < 0.005 mm^3^) and more midsize volumes (0.005 mm^3^ < V < 0.015 mm^3^).

Very similar tendency occurred for pore surfaces; however, the influence of biopolyol was stronger for I_ISO_ = 300 (Figure 6). This clearly indicates that the pore shape changed (the volumes remain similar, while surface decreased—though the sphericity increased).

**Figure 7:**
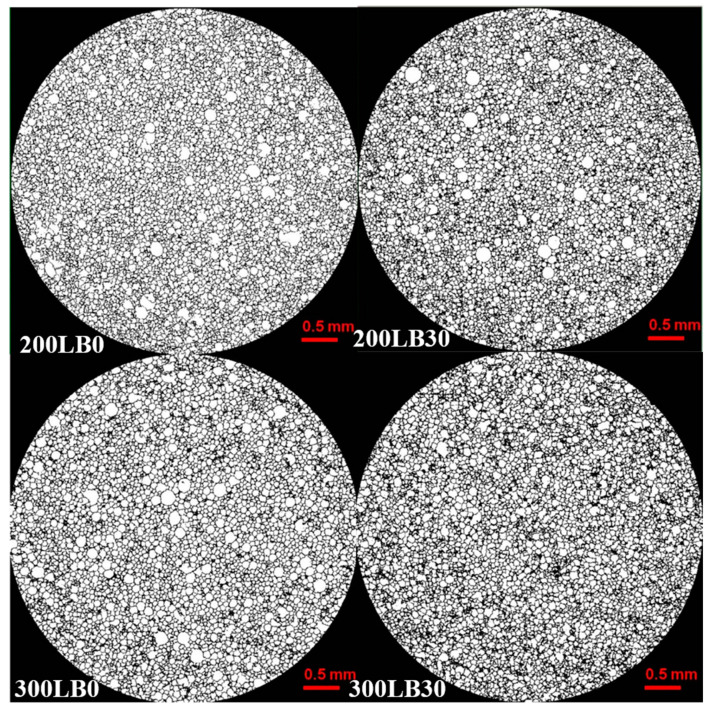
Midsection microCT scans after reconstruction and after watershed operation of anisotropic diffusion of specimens.

The pore structure is also presented in horizontal sections in Figure 7, where, in particular, the pore diameter changes may be observed. The presence of biopolyol always decreased the diameters. It is more clearly visible for foams with I_ISO_ = 300. Again, these images correspond with previously presented quantitative results of pore volumes and surface.

**Figure 8:**
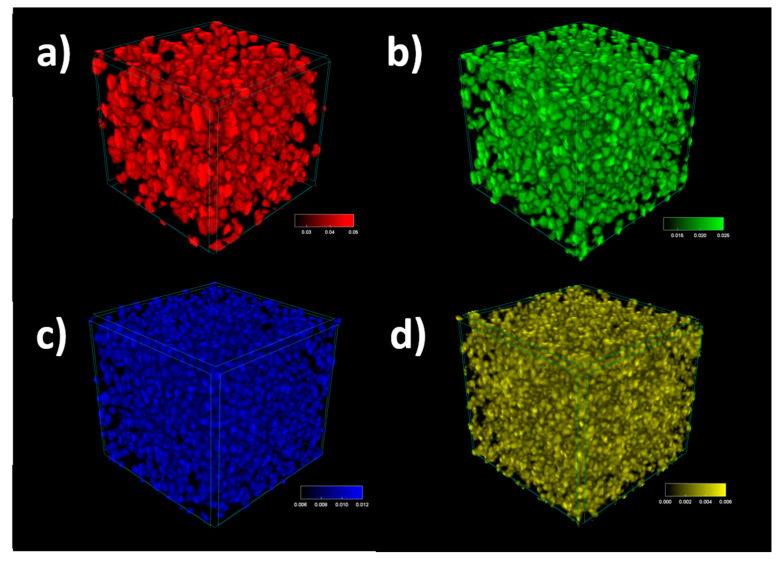
Pore distribution for specimen 300LB30, with the pore diameter of (**a**) *d* > 25 μm, (**b**) 25 μm > *d* > 12 μm, (**c**) 12 μm > *d* > 6 μm and (**d**) *d <* 6 μm.

Even though the histograms of pores distribution suggest that the fine pores constitute the majority of the specimen, in fact, the cumulated large pores volume is greater. This dependency is clearly visible for pores grouped according to their diameters (Figure 8).

### 3.2. Influence of Microstructure on Macroscopic Properties

Depending on the apparent density, rigid polyurethane foams may have different properties, so it is important to analyze materials with similar apparent density. In order to obtain materials with a similar apparent density, with the increase of the I_ISO_, a larger amount of blowing agent (*n*-pentane) and a catalyst responsible for the formation of isocyanurate rings were added to the systems. The apparent density of foams in the range 49.2–52.1 kg/m^3^ was obtained. Table 3 presents the apparent density, average values of pore diameter (SEM and microCT), thermal conductivity coefficient and thermal resistance of rigid PU foams.

**Figure 9:**
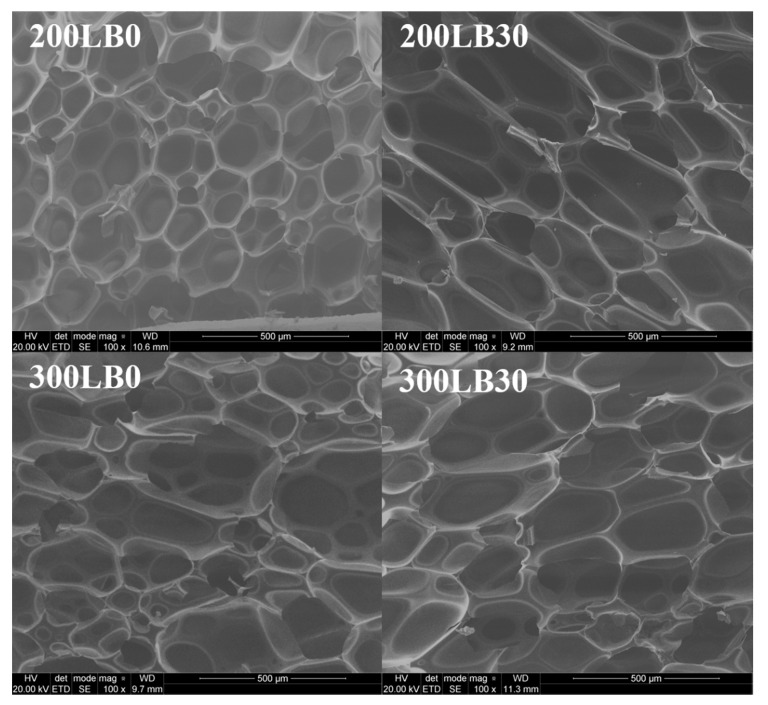
SEM of rigid polyurethane foams.

The change in pore diameter for foams containing 30 wt.% LB biopolyol compared to the foams obtained from the petrochemical polyol is not large and falls within the error limit. The larger isocyanate index caused a slight increase in the pore diameter, which could have been due to the greater amount of blowing agent used. The introduction of LB biopolyol into foam formulations resulted in changes in the shape of the cells, as shown in Figure 9.

For foams with I_ISO_ = 200 together with the increase of LB biopolyol content in the formulation, the cells were observed to grow towards the foam growth, which could have been caused by a higher temperature during synthesis [55]. Foams containing the LB biopolyol obtained at I_ISO_ = 300 showed a similar cell shape compared to the foam obtained from the petrochemical polyol, which could be caused by a relatively small amount of biopolyol in the foam mass (max 30 wt.%). Hejna et al. [56] noted that significant changes in the shape of cells are only observed for about 50 wt.% content of biopolyol obtained from waste glycerol. The pore diameter values determined using µCT differ from those obtained using SEM. The differences in these values may result from:

- more pores analyzed by the µCT technique (10000), compared to SEM (100);
- incorrect pore division in anisotropic diffusion process in the case of µCT.

The results from SEM indicate that the addition of LB biopolyol to foam formulations causes an increase in pore diameter, and the results obtained from µCT suggest an inverse relationship. However, it should be noted that, using micro CT technique, the differences in pore diameters are small and fall within the error range. This could be partially caused by the anisotropic diffusion image processing.

Table 3 also presents thermal insulation properties of foams, which are described in the form of thermal conductivity coefficient (*λ*) and thermal resistance (Equation (1)). These parameters determine the potential application possibilities of PU foams as thermal insulation materials.

In the case of PU foams, the following factors enter the value of the coefficient *λ*_gas_, *λ*_PU_, *λ*_radiation_ and *λ*_convection_ [57]. Analyzing the composition of the entire volume of PU foam, it can be noted that in the case of materials with lower apparent density the most important parameter affecting the *λ* value is the gas contained in the pores (more precisely *λ*_gas_). Parameters affecting the thermal conductivity coefficient are also the shape and content of closed cells. It has been found that as the size of the cells increases, the value of the coefficient *λ*. The type of cells (closed or open) affects the convective heat transfer in the foam. The increase in the content of closed cells limits the heat movement hindering gas exchange between volatile hydrocarbons and air, so for materials with a high content of closed cells the conduction coefficient associated with convection is omitted [58]. With the increase of LB biopolyol content, a reduction in the *λ* coefficient of 0.0024 W/(m·K) and 0.0013 W/(m·K) was observed, respectively for foams with an isocyanate index of 200 and 300. The largest decrease in the value of the coefficient *λ* was observed for I_ISO_ = 200, which may have been caused by an increase in cell dimensions that was filled with a blowing agent with a low coefficient of *λ* = 15 W/(m·K).

## 4. Conclusions

The presented results indicate that the addition of biopolyol obtained in the liquefaction process of biomass from the Baltic Sea (LB) to rigid polyurethane foams allows obtaining materials with improved strength and thermal resistance comparing to petrochemical foams. Rigid polyurethane foams obtained using 30 wt.% LB biopolyol were characterized by a similar apparent density compared to foams without biopolyol. Therefore, the materials properties changes were achieved by internal structure modification due to formulation containing biopolyol. MicroCT was an effective tool for the study of the foams’ internal structure due to very high scanning resolution and small rotation step with parallel implementation of postprocessing algorithms of whiter shade separation in 3D. The addition of biopolyol and higher I_ISO_ influenced the shape and size of the pores. PU foams with biopolyol had smaller average pores diameter for both I_ISO_ and more elongated shape compared to petrochemical foams. This was most probably caused by higher synthesis temperature. Moreover, for the higher I_ISO_, the average pore diameter increased, which was caused by a larger amount of blowing agent in the formulation. The lowest coefficient of heat transfer was characterized as 0.0246 W/m^2^K for foam 200_LB30.

## Figures and Tables

**Table 1:** Selected properties of polyols used to obtain polyurethane (PU) foams.

Polyol	L_OH_ (mg_KOH_/g)	η (mPa·s)	ρ (g/cm^3^)
LB	650	2236	1.21
Rokopol^®^RF551	440	3000–5000	1.06

**Table 2:** The compositions of rigid polyurethane foams.

Raw Materials (pbw)	Foam Symbol			
	200LB0	200LB30	300LB0	300LB30
Rokopol RF551	100	70	100	70
Biopoliol LB	0	30	0	30
AC	0.5	0.5	0.5	0.5
Dabco 15K	0.5	0.5	1	1
Dabco 33LV	0.5	0.5	0.5	0.5
DBTDL	0.5	0.5	0.5	0.5
TEGOSTAB B 8465	6	6	6	6
TCCP	10	10	10	10
*n*-pentane	12.5	12.5	20	20
pMDI	203.6	237.0	305.3	355.4

**Table 3:** Average pore diameter values and thermal insulation properties of the obtained foams.

Foam Symbol	Apparent Density (kg/m^3^)	Pore Diameter SEM (µm)	Pore Diameter MicroCT (µm)	Coefficient of Thermal Conductivity (W/m·K)	Thermal Resistance (d = 0.02 m) (m^2^·K/W)
200_LB0	49.2 ± 1.8	147 ± 26	117 ± 21	0.0270	0.741
200_LB30	49.6 ± 1.3	179 ± 25	108 ± 22	0.0246	0.810
300_LB0	49.9 ± 2.2	176 ± 30	131 ± 25	0.0261	0.767
300_LB30	52.1 ± 1.2	191 ± 38	124 ± 17	0.0248	0.806
